# Melanization slows the rapid movement of fungal necromass carbon and nitrogen into both bacterial and fungal decomposer communities and soils

**DOI:** 10.1128/msystems.00390-23

**Published:** 2023-06-20

**Authors:** François Maillard, Talia J. Michaud, Craig R. See, Lang C. DeLancey, Steven J. Blazewicz, Jeffrey A. Kimbrel, Jennifer Pett-Ridge, Peter G. Kennedy

**Affiliations:** 1 Department of Plant and Microbial Biology, University of Minnesota, St. Paul, Minnesota, USA; 2 Department of Ecology, Evolution, and Behavior, University of Minnesota, St. Paul, Minnesota, USA; 3 Physical and Life Sciences Directorate, Lawrence Livermore National Laboratory, Livermore, California, USA; 4 Life & Environmental Sciences Department, University of California Merced, Merced, California, USA; Tufts University, Medford, Massachusetts, USA

**Keywords:** bacteria, fungi, soil carbon and nitrogen cycling, forests, quantitative stable-isotope probing

## Abstract

Microbial necromass contributes significantly to both soil carbon (C) persistence and ecosystem nitrogen (N) availability, but quantitative estimates of C and N movement from necromass into soils and decomposer communities are lacking. Additionally, while melanin is known to slow fungal necromass decomposition, how it influences microbial C and N acquisition as well as elemental release into soils remains unclear. Here, we tracked decomposition of isotopically labeled low and high melanin fungal necromass and measured ^13^C and ^15^N accumulation in surrounding soils and microbial communities over 77 d in a temperate forest in Minnesota, USA. Mass loss was significantly higher from low melanin necromass, corresponding with greater ^13^C and ^15^N soil inputs. A taxonomically and functionally diverse array of bacteria and fungi was enriched in ^13^C and/or ^15^N at all sampling points, with enrichment being consistently higher on low melanin necromass and earlier in decomposition. Similar patterns of preferential C and N enrichment of many bacterial and fungal genera early in decomposition suggest that both microbial groups co-contribute to the rapid assimilation of resource-rich soil organic matter inputs. While overall richness of taxa enriched in C was higher than in N for both bacteria and fungi, there was a significant positive relationship between C and N in co-enriched taxa. Collectively, our results demonstrate that melanization acts as a key ecological trait mediating not only fungal necromass decomposition rate but also necromass C and N release and that both elements are rapidly co-utilized by diverse bacterial and fungal decomposers in natural settings.

**IMPORTANCE:**

Recent studies indicate that microbial dead cells, particularly those of fungi, play an important role in long-term carbon persistence in soils. Despite this growing recognition, how the resources within dead fungal cells (also known as fungal necromass) move into decomposer communities and soils are poorly quantified, particularly in studies based in natural environments. In this study, we found that the contribution of fungal necromass to soil carbon and nitrogen availability was slowed by the amount of melanin present in fungal cell walls. Further, despite the overall rapid acquisition of carbon and nitrogen from necromass by a diverse range of both bacteria and fungi, melanization also slowed microbial uptake of both elements. Collectively, our results indicate that melanization acts as a key ecological trait mediating not only fungal necromass decomposition rate, but also necromass carbon and nitrogen release into soil as well as microbial resource acquisition.

## INTRODUCTION

Understanding of how carbon (C) enters and leaves soil, the largest active terrestrial C pool (1), represents a key research priority due to rising global temperatures associated with increasing atmospheric carbon dioxide concentrations (2). Micro-organisms play a direct role in C retention in a wide range of terrestrial ecosystems through their inputs of dead cells (also known as necromass), which form a significant fraction of the soil organic C pool, most notably within the mineral-associated organic matter fraction (3 - 5). Of the microbial necromass compounds found in mineral-associated organic matter, those of fungal origin appear to be two- to threefold more abundant than those from bacteria (6, 7). As such, a better understanding of the post-senescent fate of fungal mycelium (fungal necromass) is particularly important in determining how organic C may persist in soils over long time periods (e.g., decades to millennia).

The decay of fungal necromass consists of two stages; an initial exponential loss of mass occurring on the order of days to weeks (8, 9), followed by an asymptotic stage that lasts from months to years (10, 11). Although this overall pattern is observed across a range of fungal necromass types (both within and across species) and hyphal morphologies, the rates of initial (*k*) and the asymptotic (*A*) mass loss are both strongly influenced by the initial biochemical composition of fungal necromass (12, 13). Specifically, amounts of nitrogen (N) as well as cell-soluble C present in newly senesced fungal necromass are both positively associated with the first stage of mass loss, while the amount of melanin, an aromatic structural compound that is resistant to decomposition (14), is positively linked with mass remaining during the later asymptotic stage (10, 13, 15). Although the influence of biochemical variation on necromass mass loss has been well demonstrated in multiple ecosystems (9, 10, 15, 16), how it impacts C and N movement from fungal necromass to soil during decomposition remains critically unquantified.

Due to the complex aromatic structure of melanin, which requires oxidative enzyme activity to degrade (17), C release from necromass is likely slowed in its presence, particularly during the later stages of decomposition where it makes up a larger proportional fraction of the remaining necromass (8, 10). Furthermore, because melanin can chemically complex with N-rich components of fungal cell walls, such as chitin (8, 18), fungal necromass with a higher melanin content may have a slower N release into soil. Notably, See et al. (13) showed that within the first phase of decomposition, most fungal necromass types exhibited a net release of N, except for highly melanized hyphae. This is in contrast to plant-derived residues, which often act as net N sinks until the late stages of decomposition (19). This phenomenon may be associated with the lower C:N ratio of fungal necromass compared with plant residues, suggesting that microbial decomposers might rapidly mineralize rather than immobilize organic N from fungal residues.

The microbial communities associated with decaying fungal necromass are co-dominated by a diverse array of bacteria and fungi (9, 10, 16, 20, 21). Within both groups, trends of early colonization by copiotrophic taxa are common due to the many labile non-structural compounds in necromass that microbes can target, including polysaccharides (13) and proteins (22) as well as structural compounds such as chitin and chitosan (23 - 25). Later colonization typically shifts to bacterial and fungal taxa with a more diverse range of ecologies (11, 16, 21), paralleling changes in fungal necromass chemistry during decomposition (e.g. declining N and increasing aromatic content over time [8]). Fernandez and Kennedy (16) demonstrated that melanization can significantly impact the structure of fungal necromass decomposer communities, with high melanin slowing the rate of succession between fast-growing and slow-growing fungi and also favoring greater abundance of bacterial taxa with complex plant cell wall degradation capacities. The extent to which these patterns corresponded with actual use of necromass C and N by fungi and bacteria, however, could not be quantified with amplicon-sequencing alone.

Stable-isotope probing (SIP) has been instrumental in determining which micro-organisms actually assimilate fungal necromass C, with a broad range of bacteria and fungi becoming significantly enriched when grown on ^13^C-labeled necromass in both laboratory (24, 26) and field settings (27). Based on the C utilization patterns in a 21-d laboratory incubation, Lopez-Mondejar et al. (24) concluded that fungal necromass C is primarily degraded by bacteria and that fungi play a minor role, being instead more important in the degradation of plant tissues. Due to notable changes in necromass chemistry over the course of fungal necromass decomposition (8), however, it seems important to assess C utilization at timepoints beyond the initial stage of decay. Furthermore, due to their differing stoichiometries (i.e., bacteria have consistently lower C:N than fungi [28]), bacterial degradation of necromass may be more strongly influenced by melanization than fungi. Specifically, the higher C:N of melanized fungal necromass may act as greater limitation on bacterial than fungal growth. Other isotopic-based studies have also shown that some fungi are able to specifically acquire N from fungal necromass (22). This suggests there is likely some partitioning of resources from decaying fungal necromass among microbial guilds, both within and across domains, although to date, no studies have examined bacterial and fungal C and N utilization from necromass simultaneously in either laboratory or field settings.

Working in a coniferous forest in Minnesota, USA and using low and high melanin fungal necromass types incubated within nylon mesh litter bags, we quantified the accrual of C and N derived from fungal necromass in surrounding soils as well as how much of each element was assimilated by the microbial communities growing on the decaying necromass. We assessed soil C and N inputs and microbial community C and N utilization at both earlier (7 and 14 d) and later (35 and 77 d) stages of decomposition. To estimate taxon-specific C and N enrichment for the bacterial and fungal communities growing on the decaying necromass, we used quantitative stable-isotope probing (qSIP, [29, 30]). We sought to test three hypotheses: (i) the faster decomposition of low melanin fungal necromass would result in greater C and N inputs into soil compared with high melanin fungal necromass, (ii) C and N enrichment would be higher for bacteria earlier in decomposition and on low melanin necromass, whereas fungal C and N enrichment would be more even over time and across necromass types, and (iii) most microbes would utilize both C and N from decaying necromass rather than specialize on only C or N.

## MATERIALS AND METHODS

### Fungal necromass generation

*Meliniomyces bicolor*, a mycorrhizal fungus associated with diverse host plants (31) and recently reclassified as *Hyaloscypha bicolor* (32), was grown on modified Melin-Norkrans (MMN) agar. Plugs of 5 mm from the active growing edges of the *M. bicolor* mycelia were transferred to glass flasks containing ^13^C enriched, ^15^N enriched, or unlabeled MMN broth. The sole source of C or N in the labeled treatment media was 99% enriched ^13^C glucose or 99% ^15^N ammonium chloride, respectively. An unlabeled treatment, which is necessary for the qSIP analyses detailed below, consisted of the same reagents, but with non-enriched glucose or ammonium chloride. To manipulate levels of melanization (as detailed in reference [16]), replicate flasks within each isotope treatment contained either 30 or 70 mL of MMN broth. Flasks were shaken on orbital shakers at 80 rpm at room temperature for 30 d. Fungal colonies were then harvested, rinsed in deionized water, and dried at 30°C in a drying oven. This temperature was found to be sufficient to kill the fungal biomass (21) while also minimizing any chemical transformations. Nylon mesh fungal necromass litter bags [~3 × 3 cm, 53 μm nylon mesh (Elko, Minneapolis, MN, USA)] containing all individual combinations of ^13^C, ^15^N, and unlabeled low or high melanin *M. bicolor* were constructed in the laboratory. Mesh size was selected to prevent root in-growth into the litter bags, while allowing full access for microbial colonization. Approximately 30 mg of dried necromass was placed in each litter bag and then closed via heat-sealing (American International Electric, City of Industry, CA, USA).

### Study site

The field incubation was conducted at the Cedar Creek Ecosystem Science Reserve in Minnesota, USA in a monodominant eastern white pine (*Pinus strobus*) forest stand (45.42577 N, 93.20852 W). The study site is characterized as a secondary forest, with *P. strobus* trees approximately 20–40 yr old and an understory composed of sparse shrubs in the genera *Prunus* and *Amelanchier*. Climatic conditions are considered continental, with a mean annual temperature of 8°C and a mean annual precipitation of 810 mm. The soils are classified as frigid Udipsamments, with a poorly developed 3–5-cm O-horizon. They are very sandy and contain 4.07% ± 1.72% C and 0.37% ± 0.14% N. At the study site, two 5 × 5 m plots were established ca. 50 m apart in early August 2018. Necromass litter bags were incubated at the litter/soil interface within each plot, each bag was separated from all other bags by 0.5 m. The bags were retrieved after 7, 14, 35 and 77 d. Additionally, bags containing each combination of labeled or unlabeled low or high melanin necromass were taken to the field, placed on the soil, but then immediately harvested to account for any mass loss associated with transport or isotopic transfer into soil during the burial process (none was detected). In total, 54 bags were incubated (two necromass types × three isotope treatments × two plots × one to two replicates/plot × four collection timepoints, see Table S1 for full details of sample replication across all variables). At each collection timepoint, the necromass bags were brought back to the laboratory on ice, where upon each bag was opened, and the remaining necromass was dried for 48 h at 30°C. Due to the limited mass, necromass dried quickly, and this low drying temperature was chosen based on previous analyses showing no significant effect on microbial community composition compared with other preservation methods (33). Fully dried fungal necromass was weighed to determine the remaining dry mass and then stored at −20°C before molecular analyses.

### Chemical analyses

Along with the necromass litter bags, soils located directly underneath each litter bag were collected at each timepoint (including day 0). Soils were collected to a depth of 5 cm using sterile 14 × 9.5 × 8 cm plastic containers (total volume ~340 cm^3^). In the laboratory, roots were removed, and soils were dried at 60°C for 48 h. Approximately 25-mg aliquots of ground soil were weighed into aluminum foil tins and analyzed for C and N on a Carlo Erba NC2100 elemental analyzer interfaced to a Thermo Electron gas isotope-ratio mass spectrometer at the Colorado Plateau Stable Isotope Laboratory located at Northern Arizona University.

To better understand the soil C and N changes associated with fungal necromass decomposition, we also conducted a laboratory chemical analysis of day 0 high and low melanin necromass. Due to a storage error, only a single replicate sample of each type was available. We used a forage fiber analysis procedure, which is commonly used for plant tissues in ecosystem studies (e.g., reference 34) and has been previously used to characterize fungal necromass (13). Briefly, cell-soluble contents (e.g., simple carbohydrates, lipids, soluble proteins, and non-protein-N; hereafter referred to as the cell-soluble fraction) were determined as the amount of mass lost after agitation in a neutral detergent solution for 2 h at 100°C. The remaining tissue was then extracted with a strong acid (72% H_2_SO_4_) for 3 h at room temperature with intermittent agitation. The mass remaining after the strong acid extraction (hereafter referred to as the non-hydrolyzable fraction) is thought to be composed of cell wall melanins and proteins complexed within them (13, 35). After each of the two chemical incubations and for the initial necromass, a subsample was taken and analyzed for total C and N content by dry combustion (Costech Analytical Technologies, Santa Clarita, CA, USA). The lack of replicate samples precluded measures of variance and statistical analyses of the fraction masses as well as C and N content.

### Molecular analyses

Due to the very low remaining mass following incubation, the low melanin samples at day 77 were excluded from molecular analyses. Additionally, because our analyses were focused on the microbial communities growing onto the fungal necromass during the incubation, day 0 necromass samples were also excluded. DNA was extracted from all other necromass samples using a phenol/chloroform protocol (36). Sample extractions were pooled between plots for each unique necromass type × isotopic label × time combination (*N* = 23) to have sufficient DNA for SIP density separation. While this pooling prevents any tests of block effects, fungal necromass microbial communities have been consistently observed to be a distinct subset of the larger soil microbial community (9, 20), suggesting that between-plot variation was not likely to be high. Samples were density fractionated in a cesium chloride density gradient formed by physical density separation in an ultracentrifuge as previously described (37) with minor modifications. For each pooled sample, 1 µg of DNA in 150 L 1× Tris-EDTA (TE) was mixed with 1.00 mL gradient buffer and 4.60 mL CsCl solution (1.887 g mL^−1^) with a final average density of 1.730 g/mL. The solution was spun in ultracentrifuge tubes (Beckman Coulter Quick-Seal, 13 × 51 mm) in an Optima L-90K ultracentrifuge (Beckman Coulter, Brea, CA, USA) using a VTi65.2 rotor at 44,000 rpm [176,284 average relative centrifugal force (RCF_avg_)] at 20°C for 109 h, with maximum acceleration and braking of the rotor to maintain the integrity of the density separations. The gradient was then separated initially into 18–33 fractions using a syringe pump delivering light mineral oil at 0.25 mL/min through a 25-G needle inserted through the top of the tube to displace the gradient solution out through a side port needle inserted through the bottom of the tube. These fractions were subsequently composited into nine fractions based on density, with the lowest composite fraction ranging from 1.665 to 1.1685 g/mL and the highest composite fraction ranging 1.765–1.784 g/mL. The density of each fraction was measured using an AR200 digital refractometer (AMETEK Reichert, Depew, NY, USA) fitted with a prism covering to facilitate measurement from 5 µL volumes, as previously described (38). DNA in each fraction was purified and concentrated using glycogen/polyethylene glycol (PEG) precipitations followed by an ethanol washing as described previously (37). Each fraction was suspended in 40 µL of 1× TE and stored at −80°C.

The microbial communities in non-fractionated and each of the nine fractions of each sample were identified using amplicon high-throughput sequencing. For bacteria, the V4 region of the 16S rRNA gene was amplified with the 515 F-806R primer pair (39). For fungi, the internal transcribed spacer region (ITS2) of the fungal rRNA operon was amplified with the 5.8S-Fun-ITS4-Fun primer pair (40). Samples were first amplified in individual 20 µL reactions containing 10 µL of Phusion Hot Start II High-Fidelity PCR Master Mix (Thermo Scientific, Waltham, MA, USA), 0.5 µL of each 20 mM primer, 1 µL of DNA template, and 8 µL of PCR-grade water. Thermocycling conditions were as follows: (i) 98°C for 30 s, (ii) 98°C for 10 s, (iii) 55°C for 30 s, (iv) 72°C for 30 s, repeat steps ii–iv for 29 times, (v) 72°C for 10 min, and (vi) infinite hold at 4°C. If initial PCRs were not successful, dilutions were performed. For all samples with amplicons, a second 10-cycle PCR was run under equivalent thermocycling conditions to add unique Golay barcodes and sequencing adaptors. PCR products were then cleaned using Ampure beads at 0.9× ratio. Each sample was pooled at equimolar concentration and sequenced on a full MiSeq lane (2 × 300 bp V3 Illumina chemistry) at the Lawrence Livermore National Laboratory, Livermore, CA, USA.

Sequences were processed using the AMPtk pipeline v1.4.2 (41). First, primers were removed, and all sequences were trimmed 250 bp (due to lower ending quality of the *R2* reads). Forward and reverse reads were then paired using VSEARCH (42). Next, sequences were denoised with UNOISE3 (43) and clustered into unique operational taxonomic units (OTUs) at 97% similarity using USEARCH (44). A 0.5% read count total cutoff was applied to each sample to eliminate very low abundance OTUs that were likely spurious due to index bleed. Taxonomy was then assigned using a hybrid algorithm that integrates results from a USEARCH global alignment against the RDP (bacteria) and UNITE (fungi) databases with both UTAX and SINTAX classifiers. Bacterial OTUs were also assigned trophic modes based on Li et al. (45), while trophic mode assignments for fungi were made using both FUNGuild (46) and FungalTraits (47).

### QSIP calculations

Quantitative stable-isotope probing was used to estimate ^13^C and ^15^N atom fraction excess (AFE) for each OTU. The qSIP method relies on tracking the shift in density, calculated by coverage in the different fractions, between unlabeled and labeled samples. An OTU is considered enriched if the density in the heavy isotope samples is significantly higher than in the light isotope samples. This change in density is used to quantify the percent enrichment (also known as atom fraction enrichment) from 0% enriched (i.e., the density expected if the OTU’s DNA contains only natural abundance ^12^C and ^14^N isotope ratios) to 100% enrichment (i.e., the density if all C or N in the OTU DNA sequence is replaced with ^13^C or ^15^N). We quantified AFE values of bacterial and fungal OTUs following a modified version of the procedure described by Hungate et al. (29) and Koch et al. (30), using average DNA concentration to normalize relative abundance of taxa within each density fraction (48, 49). We then normalized all AFE values to a maximum AFE value of 99% (the highest enrichment possible based on mass-based calculations of growth media conditions) and only retained OTUs with AFE values greater than 10% of the normalized AFE—a conservative cutoff that is approximately two to three times higher than used in other qSIP studies (2.5–5.8; [29, 50]).

### Statistical analyses

To assess the effects of melanin content, time, and their interaction on fungal necromass mass loss, we ran a two-way fully factorial analysis of variance (ANOVA). Ahead of the ANOVA, the percent mass loss values were natural log transformed to reduce heterogeneity. To assess the effects of melanin content, time {here the first two and last two sampling timepoints were combined into “earlier” and “later” groupings given their similarities in mass loss, i.e., day 7 and day 14 samples occurred during the rapid phase of decay, while day 35 and day 77 samples occurred during the slower phase of decay [see See et al. (13), for demonstration of the generality of this decay pattern across fungal necromass types], respectively} and their interaction on amounts of ^13^C and ^15^N detected in the soil cores taken from underneath decomposing fungal necromass, we ran a two-way fully factorial analyses of variance on each element separately. For the analyses of mass loss, ^13^C detection, and ^15^N detection, block was included in the full ANOVA models and determined to be non-significant, both as a main effect and in all interactions. The effects of fungal necromass melanin content and time (earlier vs later) on bacterial and fungal C and N enrichment were tested via multiple linear regressions on each combination of domain (bacteria or fungi) and element (C and N). To further evaluate the AFE values for co-enriched OTUs, we regressed AFE N on AFE C for bacteria and fungi separately, using a type II model regression for accounting for the different variances associated with AFE C and AFE N estimates.

To assess the structure of the active microbial communities colonizing the decomposing fungal necromass, we analyzed the OTUs considered enriched based on the qSIP analyses in the non-density fractionated portion of each sample (*n* = 23), as previously done in other comparable qSIP analyses (49). The bacterial and fungal datasets were first rarefied to sequence read depths of 20,000/sample and 11,000/sample, respectively [one sample with much lower total bacterial sequence reads (<5,000) was excluded; see Table S2 for final sample replication details]. Next the OTUs for each domain were partitioned into separate C-enriched and N-enriched datasets (notably, many taxa were co-enriched and therefore present in both datasets). We then applied the “adonis” and “betadisper” functions in the R package “vegan” to assess how melanin content, time, and their interactions impacted the structure of the C- and N-enriched bacterial and fungal communities. Initial permutational multivariate analysis of variance (PERMANOVA) models (i.e., those run with the adonis function) included isotope treatment (i.e., ^13^C, ^15^N, or unlabeled), but since we detected no significant effect of this variable, either as a main effect or in any interaction terms, it was removed in the final PERMANOVA models. Both analyses were based on Bray-Curtis similarities of log (*X* + 1) sequence read counts, using 9,999 permutations. Finally, to assess preferential enrichment across both necromass types and over time, indicator species analyses using the “indicspecies” package were conducted for all ^13^C- and ^15^N-enriched bacterial and fungal genera. Analyses were performed in R v4.1.2 (51).

## RESULTS

### Chemical properties of necromass

The high melanin necromass had both a higher C and lower N content than the low melanin necromass, leading to a higher initial C:N ratio (Table 1). For the cell-soluble fraction (i.e., the necromass remaining after processing with a neutral detergent solution), more mass was lost from low relative to high melanin necromass. There was also more loss of C and N, relative to initial contents, from the low melanin necromass (Table 1). By contrast, in the non-hydrolyzable fraction (i.e., the material remaining after processing with a strong acid solution), more mass, C, and N were retained in the high melanin necromass. While the C:N ratio of the cell-soluble fraction was similar between melanization levels, the C:N ratio of the non-hydrolyzable fraction from high melanin necromass was nearly double that from low melanin necromass.

**TABLE 1:** Chemical analysis of initial *M. bicolor* necromass using forage fiber (ANKOM) analysis^*a*^

Necromass type	Initial tissue			Cell-soluble fraction				Non-hydrolyzable fraction			
	% C	% N	C:N	% of initial mass	% of initial C	% of initial N	Fraction C:N	% of initial mass	% of initial C	% of initial N	Fraction C:N
High melanin	55.4	1.9	29.2	17.7	18	54.5	9.6	42.4	24.9	15.6	46.4
Low melanin	50.7	2.9	17.5	32.4	29	58.2	8.9	33.1	10.3	7.6	24.1

^*a*^ Cell-soluble fraction refers to measures following incubation in neutral detergent, while non-hydrolyzable fraction refers to measures following incubation in strong acid. See Materials and Methods section for details. Because of a sample storage error, only a single necromass sample per type was measured.

### Mass loss from necromass

**Fig 1:**
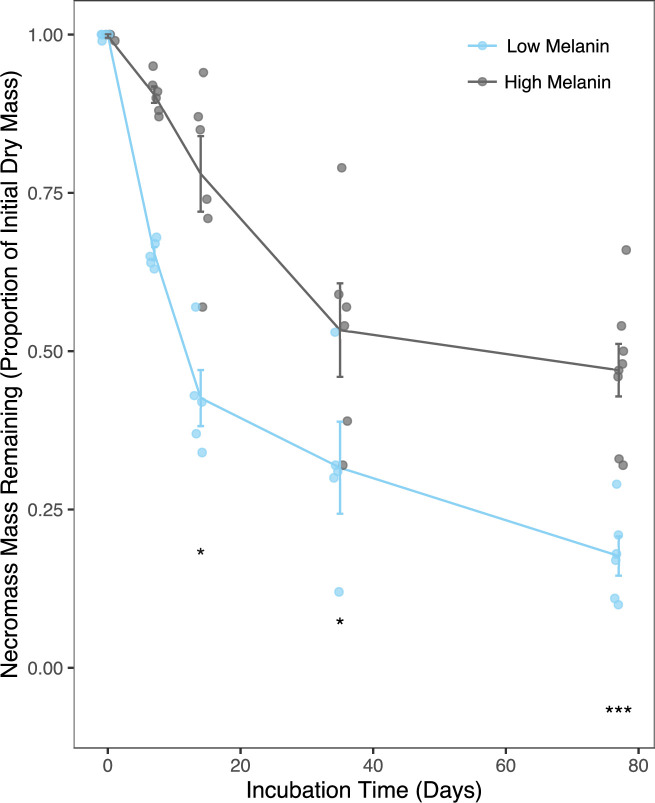
Proportion mass remaining (mean ± 1 SE) of low and high melanin *M. bicolor* necromass over the 77-d field incubation. Significance: *P < 0.05, ***P < 0.001. Total sample size for mass loss analyses, *n* = 54; however, day 0 incubation time samples were not included in the statistical models, *n* = 48.

High melanin necromass had greater mass remaining than the low melanin necromass at all timepoints during the 77-day incubation (Fig. 1). The amount of mass remaining differed significantly by necromass type (*F*_1,39_ = 60.967, *P* < 0.001) as well as over time (*F*_3,39_ = 27.099, *P* < 0.001), with the effect of melanization becoming stronger over the duration of the incubation (time × necromass type interaction: *F*_3,39_ = 3.131, *P* < 0.036). At the final sampling time, 51 ± 7% [mean ± standard error (SE)] of the high melanin necromass remained, while only 18 ± 4% of the low melanin necromass remained. There were no significant differences in mass remaining between the unlabeled and ^13^C- or ^15^N-labeled necromass treatments (*P* > 0.05, in all-pair-wise comparisons at each sampling time).

### Soil C and N inputs from necromass

**Fig 2:**
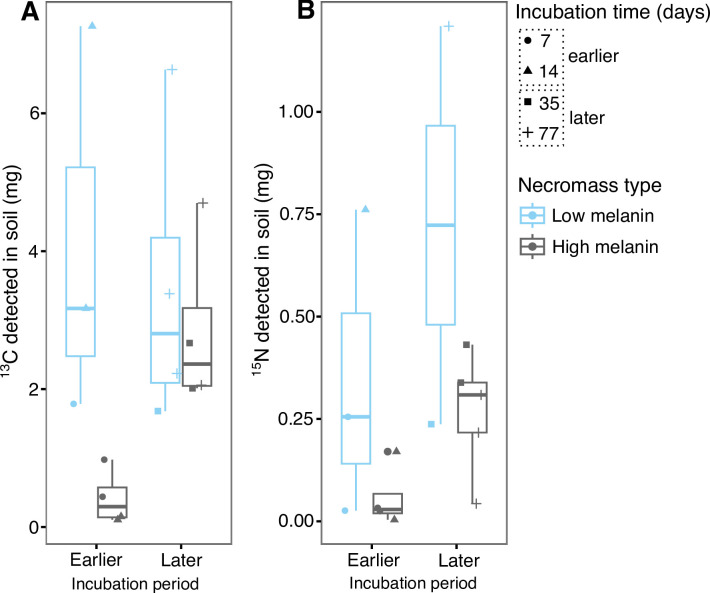
^13^C (A) and ^15^N (B) detected in soil cores taken to a depth of 5 cm directly below necromass litter bags. Values (milligram) represent the total amount of ^13^C and ^15^N detected in the entire soil core. Samples are grouped into earlier (days 7 and 14) and later (days 35 and 77) stages of decomposition. Total sample size for isotope detection analyses, *n* = 54; however, neither day 0 incubation time nor unlabeled samples were included in the statistical models, *n* = 30.

^13^C from the fungal necromass was detected in soil cores taken directly below the litter bags at all four sampling times for both necromass types (Fig. 2A). The amounts of ^13^C detected did not differ significantly between earlier and later sampling times (*F*_1,11_ = 1.222, *P* = 0.293) but did differ in a marginally significant way by necromass type (*F*_1,11_ = 4.608, *P* = 0.055), with greater but also more variable amounts of ^13^C detected in soils under low melanin necromass. There was also a trend toward greater amounts of ^13^C being detected from high melanin necromass at the later than earlier sampling times, but the interaction between necromass type and time was not significant (*F*_1,11_ = 2.576, *P* = 0.137). ^15^N from the fungal necromass was also detected in soil cores taken directly below the litter bags at all four sampling times for both necromass types (Fig. 2B). Like ^13^C, the amounts of ^15^N detected did not differ significantly by time (*F*_1,10_ = 2.861, *P* = 0.122) but did differ marginally significantly by necromass type (*F*_1,10_ = 4.872, *P* = 0.052), with greater amounts of ^15^N detected in soil under low melanin necromass. The ^15^N detection patterns were consistent by time and necromass type (time × necromass type interaction, *F*_1,10_ = 0.250, *P* = 0.628). The aforementioned trends in ^13^C and ^15^N accrual in soil were functionally equivalent when expressed as a percent initial necromass C and N (Fig. S1).

### Microbial community structure on necromass

**Fig 3:**
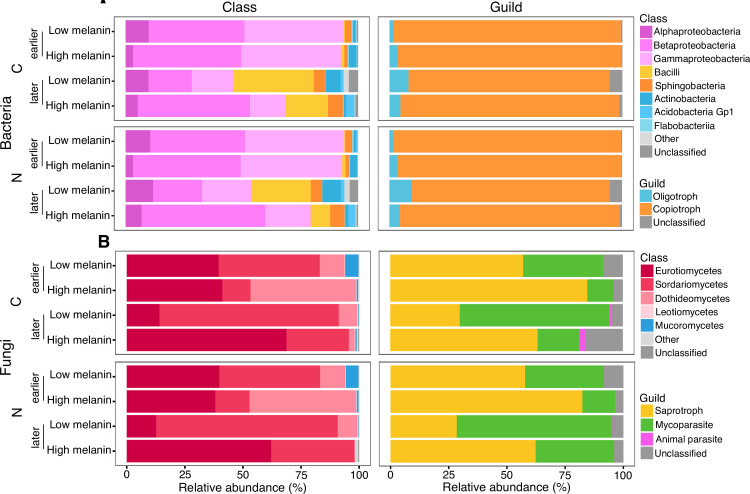
Taxonomic and guild-based composition of the enriched bacterial (A) and fungal (B) microbial communities on decomposing *M. bicolor* fungal necromass by element [carbon (C) or nitrogen (N)], incubation time (earlier and later decomposition stages), and necromass type (low and high melanin). Relative abundances are based on individual OTU sums within classes and guilds for each combination of incubation and necromass type. Very low relative abundance groups are combined into an “other” category for visual clarity. Total sample size for the quantitative stable-isotope probing (qSIP)-based microbial community analyses, *n* = 22.

The active bacterial communities present on decaying *M. bicolor* necromass consisted of a diverse array of classes, primarily alpha-, beta-, and gamma-proteobacteria as well as Bacilli, Sphingobacteria, and Actinobacteria (Fig. 3A). Alpha-, beta-, and gamma-proteobacteria were abundant in both the C- and N-enriched communities at all sampling timepoints, while Bacilli and Sphingobacteria both increased notably in abundance at later sampling timepoints. Across all timepoints, both the C- and N-enriched bacterial communities were dominated by copiotrophs, with oligotrophs reaching highest abundance on low melanin necromass at later timepoints (Fig. 3A). The C- and N-enriched fungal communities on decaying *M. bicolor* necromass were both dominated by members of the Ascomycota, particularly the classes Sordariomycetes, Eurotiomycetes, and Dothideomycetes (Fig. 3B). Members of these three classes typically represented greater than 90% of the relative sequence reads per sample, although their ratios varied, with enriched members of the Dothideomycetes being particularly abundant on high melanin necromass at earlier timepoints and Sordariomycetes being more abundant on low melanin necromass at later timepoints. The C- and N-enriched fungal communities had similar patterns in terms of guild composition, with saprotrophs and mycoparasites being co-dominant (Fig. 3B). These guilds differed in abundance depending on necromass type, however, with saprotrophs most abundant on high melanin necromass and mycoparasites being most abundant on low melanin necromass at both earlier and later timepoints.

**Fig 4:**
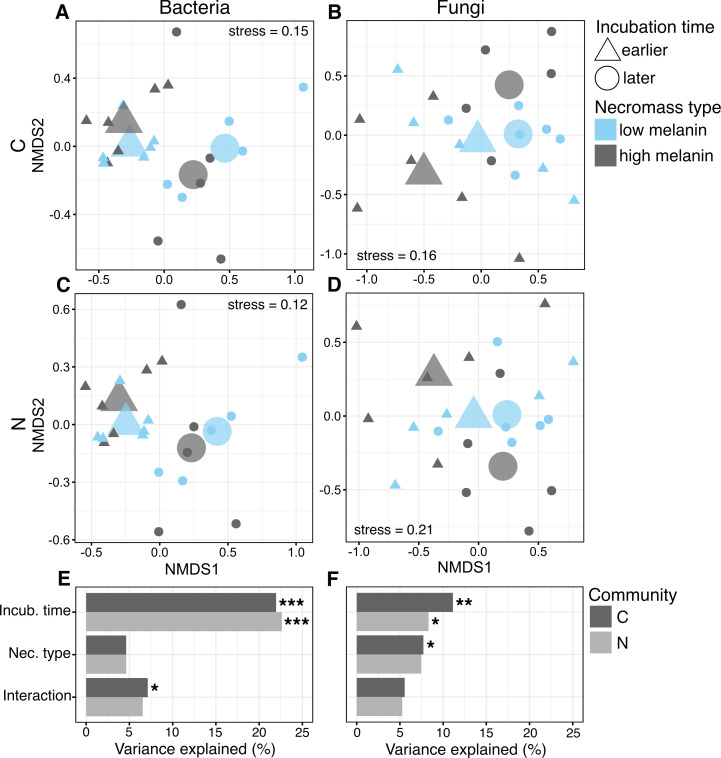
Non-metric multi-dimensional scaling (NMDS) analysis of the carbon (C) (A and B) and nitrogen (N) (C and D) enriched bacterial and fungal OTU communities on decomposing *M. bicolor* fungal necromass depending on incubation time (earlier and later decomposition stages) and necromass type (low and high melanin). Small symbols represent individual samples, and large symbols represent centroids. (E and F) Variance explained based on PERMANOVA depending on incubation time, necromass type, and their interaction. Significance: *P ≤ 0.05; **P ≤ 0.01; ***P ≤ 0.001. Total sample size for the qSIP-based microbial community analyses, *n* = 22.

The overall C-enriched bacterial communities on decaying *M. bicolor* necromass differed significantly in composition between the earlier and later timepoints (*F*_1,18_ = 5.961, *P* < 0.001, Fig. 4A) but not by necromass type (*F*_1,18_ = 1.258, *P* = 0.205). There was also a significant interaction between time and necromass type on C-enriched bacterial community composition (*F*_1,18_ = 1.935, *P* = 0.041), likely due to greater temporal variation on high than low melanin necromass. Additionally, dispersion in the composition of the C-enriched bacterial communities was significantly higher at later than earlier timepoints regardless of necromass type (*F*_1,20_ = 7.542, *P* = 0.020). The N-enriched bacterial communities followed similar trends, with significant differences in composition (*F*_1,18_ = 6.151, *P* < 0.001, Fig. 4C) and dispersion (*F*_1,18_ = 1.260, *P* = 0.222) at earlier than later timepoints but no significant differences by necromass type (*F*_1,20_ = 6.276, *P* < 0.021). For the fungi, the C-enriched communities on decomposing *M. bicolor* necromass differed significantly in composition between the earlier and later timepoints (*F*_1,18_ = 2.651, *P* = 0.003, Fig. 4B) but also by necromass type (*F*_1,18_ = 1.838, *P* = 0.024). Furthermore, dispersion in composition of the C-enriched fungal communities was significantly higher on high than low melanin necromass (*F*_1,20_ = 5.603, *P* = 0.028). Finally, for the N-enriched fungal communities, there were only significant differences in composition or dispersion between earlier and later timepoints (*F*_1,18_ = 1.897, *P* = 0.036, Fig. 4D).

### Microbial community uptake of necromass C and N

**Fig 5:**
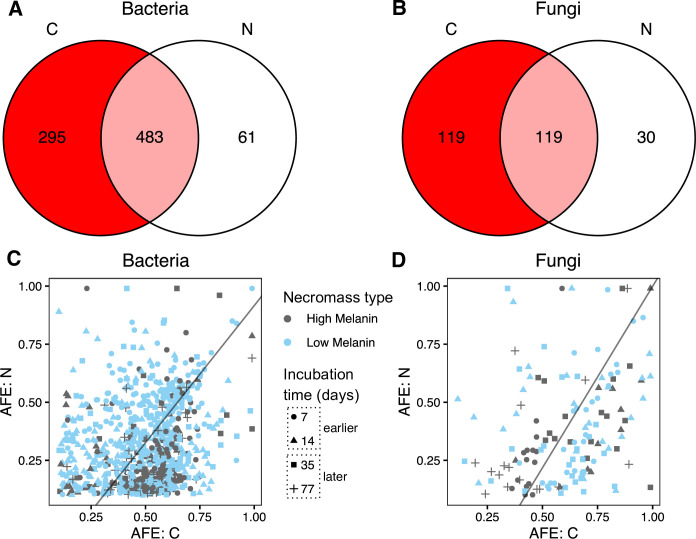
Venn diagrams of (A) bacterial OTUs and (B) fungal OTUs enriched in carbon (C), nitrogen (N), or both C and N on decomposing *M. bicolor* fungal necromass over the 77-d field incubation. Relationships between AFE ^13^C and ^15^N enrichment for co-enriched (C) bacterial and (D) fungal OTUs only. Slopes (black lines) based on model II regressions. Total sample size for the qSIP-based microbial community analyses, *n* = 22.

Of the 987 bacterial and 312 fungal OTUs detected on decaying fungal necromass, 839 (93%) and 268 (89%) were considered enriched in C and/or N. Specifically, 778 (92%) of these enriched bacterial OTUs were enriched with ^13^C, while 238 (89%) of enriched fungal OTUs were enriched with ^13^C. On the ^15^N-labeled necromass, 554 (65%) of enriched bacterial taxa were enriched with ^15^N, while 149 (56%) of enriched fungal OTUs were enriched with ^15^N. Although more total bacterial and fungal OTUs were enriched in C than N, many bacterial and fungal OTUs were simultaneously enriched in both elements (Fig. 5A and B). For those co-enriched OTUs, there was a significant positive relationship between C and N enrichment (bacteria: *t* = 6.794, *P* < 0.001, adj. *R* ^2^ = 0.052; fungi: *t* = 4.136, *P* < 0.001, adj. *R* ^2^ = 0.103, Fig. 5C and D). The slopes for bacterial and fungal co-enrichment were similar (bacterial slope: mean = 1.19, C.I. = 0.89–1.61; fungal slope: mean = 1.56, C.I. = 0.99–2.73), with both having *x*-intercepts above zero.

**Fig 6:**
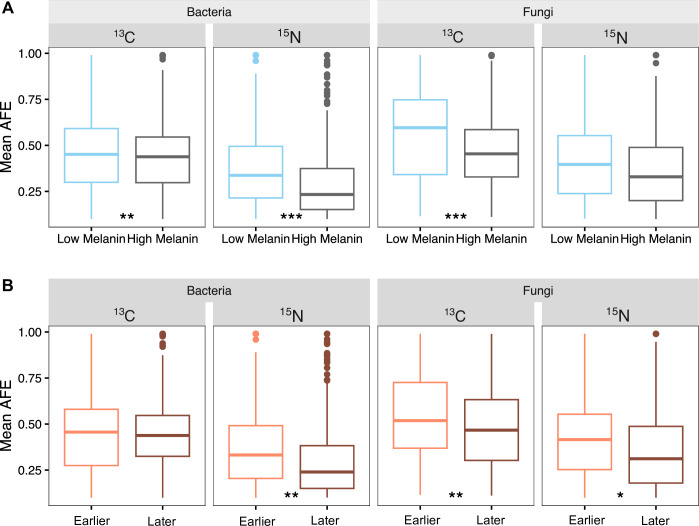
AFE of carbon (^13^C) and nitrogen (^15^N) enrichment for bacterial and fungal communities present on decomposing *M. bicolor* fungal necromass by (A) necromass type (low and high melanin) and (B) incubation time (earlier and later decomposition stages). Significance: *P ≤ 0.05; **P ≤ 0.01; ***P ≤ 0.001. Total sample size for the qSIP-based microbial community analyses, *n* = 22.

At the community level, bacteria became significantly more enriched (i.e., had higher ^13^C and ^15^N AFE) on low melanin necromass relative to high melanin necromass (C: *t* = 2.757, *P* = 0.006; N: *t* = 4.797, *P* < 0.001, Fig. 6A; Fig. S3). While the mean ^13^C AFE of bacterial communities did not change significantly over time (*t* = 0.979, *P* = 0.328), mean bacterial ^15^N AFE was significantly lower at the later timepoints (*t* = −3.292, *P* = 0.001, Fig. 6B). For the fungal community, ^13^C AFE was also significantly higher for low melanin necromass (*t* = 3.387, *P* < 0.001), but ^15^N AFE was not significantly different between necromass types (*t* = 0.522, *P* = 0.602, Fig. 6A). With time, the average AFE of fungal communities declined significantly between earlier and later sampling times, for both C (*t* = −2.778, *P* = 0.006) and N (*t* = −1.973, *P* = 0.049) (Fig. 6B). We reiterate that the composition of both the ^13^C and ^15^N bacterial and fungal communities significantly changed between earlier and later sampling timepoints and also that the composition of the ^13^C-enriched fungal communities differed significantly between low and high melanin necromass (Fig. 3).

**Fig 7:**
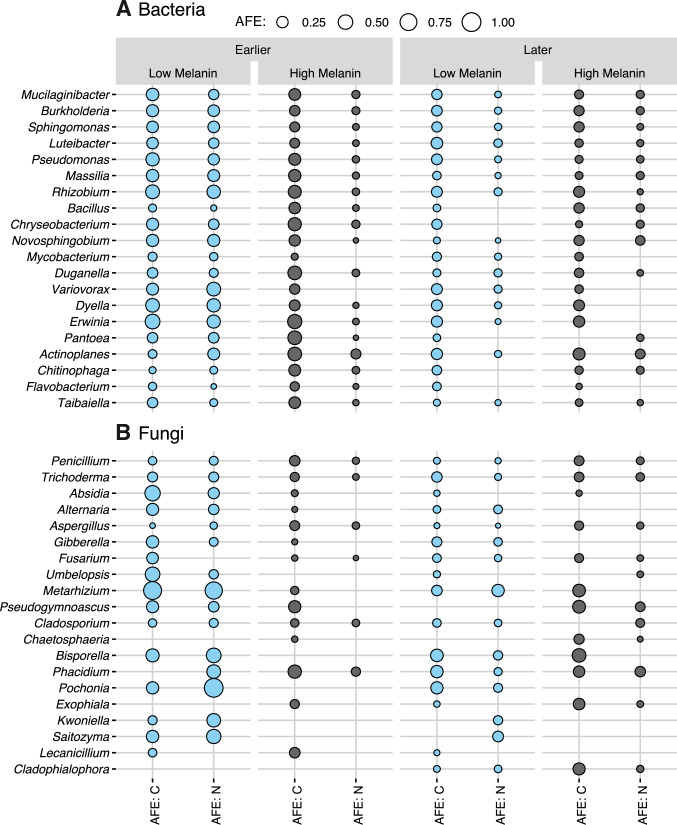
AFE in carbon (^13^C) and nitrogen (^15^N) enrichment of 20 most enriched (A) bacterial genera and (B) fungal genera on decomposing *M. bicolor* fungal necromass by incubation time (earlier and later decomposition stages) and necromass type (low and high melanin). Circles represent mean AFE, with genera sorted by AFE sums across all combinations (i.e. genera higher on the *Y* axis have greater total enrichment across all eight combinations of incubation time and necromass type than those lower on the *Y* axis). Total sample size for the qSIP-based microbial community analyses, *n* = 22.

The top five most C- and N-enriched bacterial genera across all necromass types and times were *Mucilaginibacter*, *Burkholderia*, *Sphingomonas*, *Luteibacter*, and *Pseudomonas* (Fig. 7). There was limited evidence of C or N specialization (i.e., uptake of only a single element) among the top 50 most enriched bacterial genera; rather co-enrichment was common, and AFE C and AFE N were significantly positively correlated on both necromass types at earlier and later timepoints (Fig. S2). There were a number of bacterial genera, however, that had significantly greater AFE C and/or N at earlier timepoints and/or on low melanin necromass (Table 2). Furthermore, there was no significant correlation between bacterial genera relative abundance (based on sequence read counts) and AFE C or AFE N (Fig. S3). For fungi, the top 5 most C- and N-enriched fungal genera across all necromass types and harvest times were *Penicillium*, *Trichoderma*, *Absidia*, *Alternaria*, and *Aspergillus* (Fig. 7). Only three fungal genera (*Penicillium*, *Trichoderma*, and *Aspergillus*) were enriched in both C and N on both necromass types at both time groupings. There were a number of fungal genera that had significantly greater AFE C and N on low melanin necromass but none with significantly greater AFE C and N on high melanin necromass (Table 2). Over time, unlike bacteria, no fungal genera had significantly greater AFE C or N at earlier sampling points, while two genera (*Cladophialophora* and *Dactylella*) had significantly higher enrichment at later timepoints. Like bacteria, however, there was no significant correlation between fungal genera relative abundance (based on sequence read counts) and AFE C or AFE N (Fig. S3).

**TABLE 2:** Indicator analysis of bacterial and fungal genera with preferential carbon and/or nitrogen enrichment depending on necromass type (low and high melanin) and incubation time (earlier and later decomposition stages)^*a*^

Organism type	Melanin level or incubation time	Enrichment	Organism(s)
Bacteria	Low	C enriched	*Beijerinckia, Cellulomonas, Cupriavidus, Janthinobacterium, Labrys, Luteolibacter, Methylorosula, Mycobacterium, Pandoraea, Rhizorhabdus, Rosea, Rubrivavax, Variovorax, Xanthomonas*
		N enriched	*Beijerinckia, Cellulomonas, Cellvibrio, Cupriavidas, Dyadobacter, Ewingella, Frondihabitans, Janthobacterium, Labrys, Nocardioides, Pandoraea, Phenylobacterium, Rhizorhabdus, Rosea, Rubrivavax, Stenotrophomonas, Variovorax, Xanthomonas*
	High	C enriched	*Inquilinus*
		N enriched	*Inquilinus*
Fungi	Low	C enriched	*Absidia, Alternaria, Clonostachys, Gibberella, Kwoniella, Metarhizium, Pochonia, Saitozyma, Umbelopsis*
		N enriched	*Absidia, Alternaria, Gibberella, Metarhizium, Pochonia, Saitozyma, Umbelopsis*
Bacteria	Earlier	C enriched	*Budvicia, Pedobacter, Pseudodugenella, Salmonella, Stenotrophomonas, Xanthomonas*
		N enriched	*Budvicia, Nocardioides, Pedobacter, Pseudodugenella, Salmonella, Stenotrophomonas, Xanthomonas*
	Later	N enriched	*Amycolatopsis*
Fungi	Later	C enriched	*Cladophialophora*
		N enriched	*Cladiophialophora, Dactylella*

^*a*^ Significance: *P* < 0.05 for all genera listed. Total sample.

## DISCUSSION

Collectively, this study examined multiple necromass types (low and high melanin), multiple resources (C and N), multiple incubation times (earlier and later stages of decomposition), and both soil inputs as well as bacterial and fungal utilization from fungal necromass under field conditions. Our motivations were to: (i) determine the extent to which fungal necromass melanization affected amounts of C and N release into soil at both earlier and later stages of decomposition, (ii) determine the extent to which fungal necromass melanization affected C and N acquisition by decomposer microbial communities at both earlier and later stages of decomposition, and (iii) determine the extent to which fungal necromass serves as a dual source of nutrition and energy for decomposer microbial communities. Overall, we found that melanization did have significant inhibitory effects on C and N inputs into soil as well as microbial acquisition of both resources at earlier and later stages of decomposition. Furthermore, we demonstrated that decaying fungal necromass is frequently used by bacterial and fungal decomposers as a dual source of nutrition and energy. Below we discuss each of these findings in greater detail, particularly in light of our hypotheses.

We first hypothesized that the decomposition of low melanin fungal necromass would result in greater overall C and N inputs into soil due to a faster decay rate. This was broadly supported by our data, which showed more C and N accrual in soils under the more rapidly decomposing low melanin necromass. We attribute the differences between necromass types to multiple aspects of their chemical composition. Although not specifically measured here, previous studies have shown that melanin itself is highly resistant to decay (10, 17), thus its presence in fungal cell walls likely directly lowers decay rates. Additionally, because melanin can complex with other C- and N-containing components in cell walls (8, 18), it likely also slows microbial degradation indirectly by lowering substrate resource availability. Our laboratory chemical analysis results were consistent with this mechanism, as we found that the non-hydrolyzable fraction of high melanin necromass retained three times more C and two times more N than the non-hydrolyzable fraction of low melanin necromass. Additionally, the lower initial C:N ratio of low melanin necromass, which was also observed in a previous study (16), likely makes it a broader target of decay for microbial decomposers than high melanin necromass. Both the higher OTU richness and AFE values of bacteria and fungi on low melanin necromass in the qSIP results support greater microbial activity on the low melanin necromass. While current models of long-term soil C persistence suggest that faster rather than slower microbial necromass decomposition is important for stimulating soil C accrual (52), others have shown that fungal melanization correlates positively with soil C retention (53, 54). Given these contrasting perspectives, future studies measuring how much fungal necromass C ends up in the mineral-associated fraction and in what form (e.g., is it melanin and its derivatives or C from other sources?) will be key to better understanding how melanin mechanistically influences soil C persistence.

For our second hypothesis, we speculated that bacterial and fungi would differ in their C and N enrichment patterns by both necromass type and over time. In particular, we predicted that bacterial enrichment would be greater on low melanin necromass and earlier in decomposition, which was supported by the AFE C and AFE N patterns as well as the indicator genus analyses. This finding may reflect that nearly all of the ^13^C and ^15^N enriched bacterial OTUs we identified are classified as copiotrophs and were likely targeting labile C and N sources that were comparatively more available in low melanin necromass during the initial stage of decomposition (i.e., our laboratory chemical analyses showed that low melanin necromass did have proportionately higher cell-soluble mass, C, and N than high melanin necromass). In contrast to a previous SIP-based study proposing that fungi play a limited role in fungal necromass decomposition (24), we found equivalent patterns of greater fungal enrichment on low melanin necromass as well as early in decomposition. These results suggest that in addition to being key decomposers of more complex soil organic matter inputs (24), fungi also co-contribute significantly to the initial decay of resource-rich substrates such as fungal necromass. A critical future research direction will be determining how bacterial-fungal interactions influence decomposition dynamics (55). Specifically, understanding the extent to which bacteria and fungi compete vs synergize in the depolymerization and assimilation of necromass C and N will be aided by compound-specific isotope tracing studies (25) as well as laboratory experiments directly manipulating decomposer community species composition.

Similar to previous studies, we found that there were significant shifts in bacterial and fungal community structure over time (9, 11, 15, 20, 21). This was true for both the C and N enriched bacterial and fungal communities, suggesting that the observed community shifts were not driven by limitation of a single component of decaying necromass. The compositional changes did, however, correspond with shifts in mean AFE enrichment, indicating later-colonizing decomposers do assimilate less resources from more decayed necromass, particularly lower amounts of N. This finding is broadly consistent with an earlier chemical analysis of field-incubated decaying *M. bicolor* necromass, which showed proportional increases in the ratio of both carbohydrates and aromatic compounds to N-containing compounds over time (8). Despite the overall trends at the community level, some microbial taxa with known abilities to degrade more complex molecules, including aromatic hydrocarbons (56, 57), were able to liberate resources from more recalcitrant fungal necromass, such as *Cladophialophora*, which showed preferential C and N enrichment at the later stages of decomposition, as well as *Chaetosphaeria*, which was only enriched on high melanin necromass. Arguably, the most intriguing temporal pattern was the presence of several fungal genera that are usually classified as mycoparasites (e.g. *Clonostachys*, *Metarhizium*, and *Trichoderma*) early in necromass decomposition. These results align with previous research showing these genera can be abundant during fungal necromass decomposition and may be involved in parasitizing fungi participating in the decomposition processes (9, 11). Our results could be due to rapid cross-feeding and/or parasitization but also suggest a second possibility—mycoparasitic fungi might directly participate in necromass decomposition as scavengers. While these potential scavenging mechanisms remain unknown, they might be associated with the substantial capacities of mycoparasitic fungi for fungal cell wall degradation (58 - 60).

Our third hypothesis surmised that due to relatively high labilty of the non-structural and structural compounds present in fungal necromass, it would frequently be the target of simultaneous C and N acquisition by both bacteria and fungi. We found general support for this hypothesis, with at least 50% of the enriched bacterial and fungal OTUs being co-enriched in C and N. Moreover, we documented positive overall relationships between the amounts of C and N enrichment for both bacteria and fungi, suggesting their uptake mechanisms are coupled. While we are confident that coupled C and N uptake is likely a common process in microbial utilization of fungal necromass resources, the high variation across OTUs (as shown in Fig. 5) suggests that the specific nature of the coupling likely varies considerably across taxa. Furthermore, because our labeling approach was not compound specific (e.g. reference [25]) we could not determine which C- and N-containing molecules were consumed from fungal necromass and by which microbial domain (i.e., bacteria and/or fungi). Specifically, it is unclear whether necromass decomposers utilized both C and N from the same compounds or targeted different compounds for C and N separately. Still, the positive relationships we found between C and N enrichment of bacterial and fungal genera during the early decomposition stage point out that both domains actively targeted labile C- and N-rich compounds, such as amino-sugars, nucleic acids, and proteins (24, 26, 61). Interestingly, late in the decomposition, only bacteria presented a positive relationship between C and N enrichment. Thus, we speculate that necromass-associated bacterial communities participate in the degradation of non-structural polymers such as chitin (both a C and N source), while fungi may more substantially be involved in the decomposition of glucan and mannan polymers and lipids.

Regarding the necromass used in this study, both types were likely richer in initial C and N than naturally occurring fungal necromass due to the resorptive nature of resources within fungal mycelia during senescence (62). Additionally, our necromass samples were incubated as a single physical aggregate, which is representative of necromass generated from decaying fungal sporocarps but not necessarily representative of more diffuse vegetative mycelium in soil. Given these factors, we believe our C and N soil inputs as well as levels of microbial enrichment should best be considered as maximal estimates. Furthermore, because the necromass was incubated in nylon mesh bags, we limited direct physical interactions between the decaying necromass and soil particles, which may affect both the rates of decomposition as well as the long-term retention of both C and N in soil (63). Similarly, the pore size of the bags prevented larger soil mesofauna (e.g., mites) from directly accessing the necromass, which may have influenced both the observed mass loss rates as well as microbial community dynamics (64). Finally, the lack of replication within each timepoint for each necromass type and element combination made our estimates of C and N enrichment of bacteria and fungi more variable than in other qSIP-based studies (15, 50, 65). Thus, more robust studies are needed going forward, particularly characterizing taxa with lower levels of enrichment.

### Conclusions

Using isotopic labeling, we have demonstrated that C and N release from decaying fungal necromass into soil occurs rapidly, and both elements are frequently assimilated by a diverse range of bacterial and fungal decomposers in natural settings. Furthermore, we show that initial biochemical composition of fungal necromass is a key trait that not only impacts its rates of overall mass loss, but also rates of C and N release as well as resource use by necromass-associated microbial communities. Finally, our results indicate that fungal necromass is consistently targeted by many microbial decomposers as a dual source of C and N, suggesting that future studies of fungal necromass effects on soil biochemical cycling should focus on both elements whenever possible.

## Data Availability

Bacterial and fungal sequence reads per sample are deposited in the NCBI Sequence Read Archive under the BioProject ID PRJNA916504.

## SUPPLEMENTAL MATERIAL

Fig S1 msystems.00390-23-s0001.pdf Soil C and N detection as a proportion of initial necromass.

Fig S2 msystems.00390-23-s0002.pdf Bacterial and fungal relationships between AFE C and AFE N depending on necromass type and time.

Fig S3 msystems.00390-23-s0003.pdf Bacterial and fungal relationships between AFE and sequence read counts.

TABLE S1 msystems.00390-23-s0004.pdf Fungal necromass samples for mass loss and soil carbon and nitrogen isotopic analyses.

TABLE S2 msystems.00390-23-s0005.pdf Fungal necromass samples for the qSIP and associated microbial community analyses.

ASM does not own the copyrights to Supplemental Material that may be linked to, or accessed through, an article. The authors have granted ASM a non-exclusive, world-wide license to publish the Supplemental Material files. Please contact the corresponding author directly for reuse.
