# Supplementary material for: Melanization slows the rapid movement of fungal necromass carbon and nitrogen into both bacterial and fungal decomposer communities and soils
Source: mSystems. 2023 Jun 20;8(4):e00390-23. doi: 10.1128/msystems.00390-23 (PMC10469842; doi:10.1128/msystems.00390-23)

Figure S1. Percent of initial (A) Carbon and (B) Nitrogen present in decomposing *M. bicolor* fungal necromass detected in soil cores taken to a depth of 5 cm directly below necromass litter bags. One later low melanin sample had a value above 100%, which was adjusted to 100% before plotting.

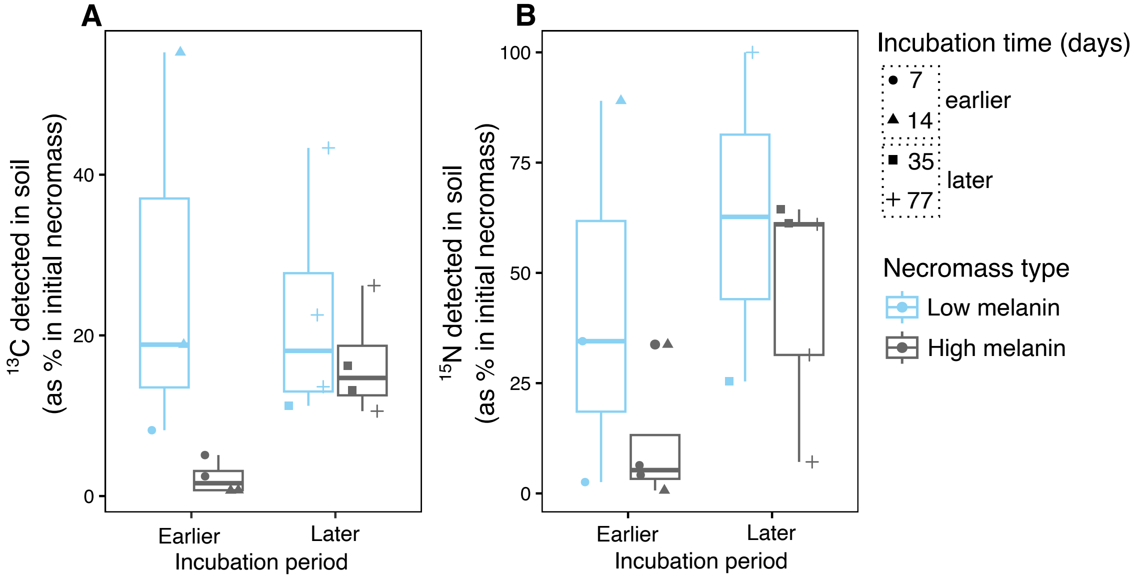

Supplement: Fig S1 — Soil C and N detection as a proportion of initial necromass. [file msystems.00390-23-s0001.pdf]
