# Supplementary material for: Melanization slows the rapid movement of fungal necromass carbon and nitrogen into both bacterial and fungal decomposer communities and soils
Source: mSystems. 2023 Jun 20;8(4):e00390-23. doi: 10.1128/msystems.00390-23 (PMC10469842; doi:10.1128/msystems.00390-23)

Figure S2. Relationship between atom fraction excess (AFE) Carbon (C) and Nitrogen (N) depending on necromass type (low and high melanin), incubation time (earlier and later stages of decomposition), and domain (bacteria (A) and fungi (B)). Points are individual genus mean AFE N versus genus mean AFE C values for all of the genera present in that specific treatment combination (e.g. early + high melanin).

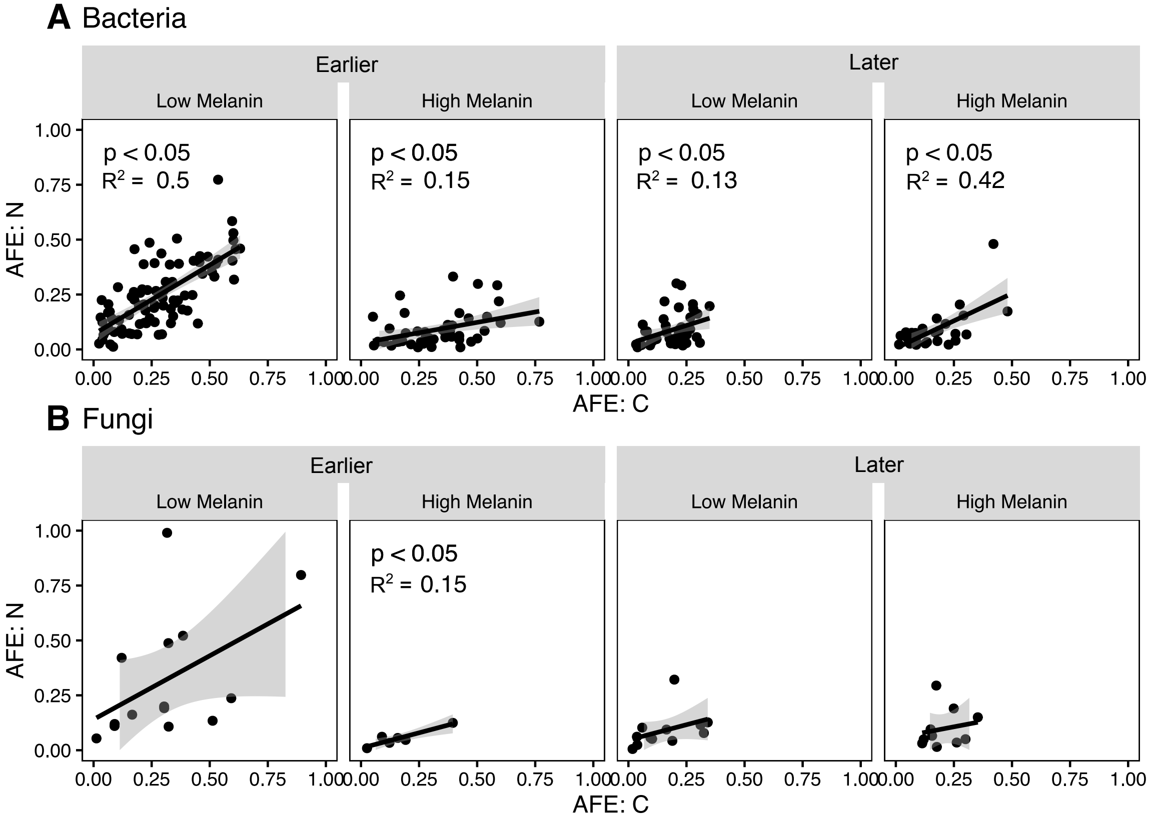

Supplement: Fig S2 — Bacterial and fungal relationships between AFE C and AFE N depending on necromass type and time. [file msystems.00390-23-s0002.pdf]
