# Supplementary material for: Melanization slows the rapid movement of fungal necromass carbon and nitrogen into both bacterial and fungal decomposer communities and soils
Source: mSystems. 2023 Jun 20;8(4):e00390-23. doi: 10.1128/msystems.00390-23 (PMC10469842; doi:10.1128/msystems.00390-23)

Figure S3.Relationship between atom fraction excess (AFE) Carbon (C) and Nitrogen (N) and natural log(X+1) sequence read counts for (A) bacteria and (B) fungi. Points represent individual genera.

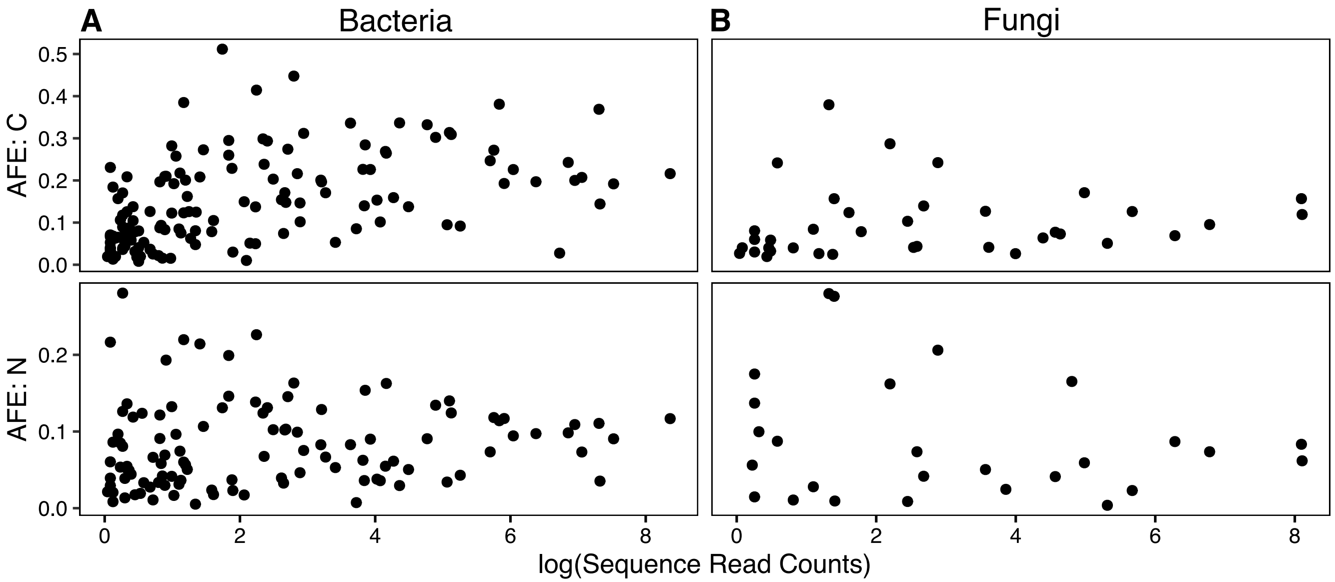

Supplement: Fig S3 — Bacterial and fungal relationships between AFE and sequence read counts. [file msystems.00390-23-s0003.pdf]
