# Supplementary material for: Melanization slows the rapid movement of fungal necromass carbon and nitrogen into both bacterial and fungal decomposer communities and soils
Source: mSystems. 2023 Jun 20;8(4):e00390-23. doi: 10.1128/msystems.00390-23 (PMC10469842; doi:10.1128/msystems.00390-23)
Supplement: TABLE S1 — Fungal necromass samples for mass loss and soil carbon and nitrogen isotopic analyses. [file msystems.00390-23-s0004.pdf]

Table S1.Fungal necromass samples for mass loss and soil carbon and nitrogen isotopic analyses.

Sample	Bag Number	Block	Necromass Type	Isotope	Harvest Day	Time
1	263	1	Low Melanin	Unlabeled	0	NA
2	312	1	High Melanin	Carbon	0	NA
3	358	2	Low Melanin	Nitrogen	0	NA
4	453	1	Low Melanin	Nitrogen	0	NA
5	595	2	High Melanin	Carbon	0	NA
6	791	2	High Melanin	Unlabeled	0	NA
7	429	1	Low Melanin	Carbon	7	Earlier
8	487	2	High Melanin	Carbon	7	Earlier
9	524	1	High Melanin	Carbon	7	Earlier
10	528	2	Low Melanin	Carbon	7	Earlier
11	533	2	Low Melanin	Nitrogen	7	Earlier
12	544	1	High Melanin	Unlabeled	7	Earlier
13	556	2	Low Melanin	Unlabeled	7	Earlier
14	559	1	Low Melanin	Nitrogen	7	Earlier
15	569	2	High Melanin	Nitrogen	7	Earlier
16	572	2	High Melanin	Unlabeled	7	Earlier
17	669	1	High Melanin	Nitrogen	7	Earlier
18	688	1	Low Melanin	Unlabeled	7	Earlier
19	386	1	Low Melanin	Unlabeled	14	Earlier
20	508	2	High Melanin	Nitrogen	14	Earlier
21	513	2	Low Melanin	Unlabeled	14	Earlier
22	539	1	High Melanin	Carbon	14	Earlier
23	543	2	Low Melanin	Nitrogen	14	Earlier
24	553	2	High Melanin	Unlabeled	14	Earlier
25	574	2	Low Melanin	Carbon	14	Earlier
26	582	2	High Melanin	Carbon	14	Earlier
27	591	1	High Melanin	Nitrogen	14	Earlier
28	604	1	High Melanin	Unlabeled	14	Earlier
29	663	1	Low Melanin	Carbon	14	Earlier
30	291	1	High Melanin	Carbon	35	Later
31	313	2	Low Melanin	Unlabeled	35	Later
32	327	2	High Melanin	Unlabeled	35	Later
33	442	1	Low Melanin	Nitrogen	35	Later
34	458	2	Low Melanin	Carbon	35	Later
35	462	1	High Melanin	Unlabeled	35	Later
36	520	1	High Melanin	Nitrogen	35	Later
37	563	2	High Melanin	Carbon	35	Later
38	565	2	High Melanin	Nitrogen	35	Later
39	607	1	Low Melanin	Unlabeled	35	Later
40	682	1	Low Melanin	Carbon	35	Later
41	254	1	Low Melanin	Carbon	77	Later
42	360	2	Low Melanin	Unlabeled	77	Later
43	361	2	High Melanin	Nitrogen	77	Later
44	366	1	High Melanin	Unlabeled	77	Later
45	370	1	High Melanin	Carbon	77	Later
46	448	2	Low Melanin	Carbon	77	Later
47	503	1	Low Melanin	Unlabeled	77	Later
48	537	1	High Melanin	Unlabeled	77	Later
49	540	1	High Melanin	Nitrogen	77	Later
50	551	2	High Melanin	Carbon	77	Later
51	571	1	High Melanin	Nitrogen	77	Later
52	576	2	High Melanin	Unlabeled	77	Later
53	578	2	Low Melanin	Carbon	77	Later
54	586	1	Low Melanin	Nitrogen	77	Later
