# Supplementary material for: Melanization slows the rapid movement of fungal necromass carbon and nitrogen into both bacterial and fungal decomposer communities and soils
Source: mSystems. 2023 Jun 20;8(4):e00390-23. doi: 10.1128/msystems.00390-23 (PMC10469842; doi:10.1128/msystems.00390-23)
Supplement: TABLE S2 — Fungal necromass samples for the qSIP and associated microbial community analyses. [file msystems.00390-23-s0005.pdf]

Table S2.Fungal necromass samples for the qSIP and associated microbial community analyses.

Sample	Lab ID number	Necromass Type	Isotope	Time	Days Incubated
1	19	High Melanin	Carbon	Earlier	7
2	1	High Melanin	Nitrogen	Earlier	7
3	7	High Melanin	Unlabeled	Earlier	7
4	25	Low Melanin	Carbon	Earlier	7
5	8	Low Melanin	Nitrogen	Earlier	7
6	20	Low Melanin	Unlabeled	Earlier	7
7	9	High Melanin	Carbon	Earlier	14
8	12	High Melanin	Nitrogen	Earlier	14
9	10	High Melanin	Unlabeled	Earlier	14
10	21	Low Melanin	Carbon	Earlier	14
11	11	Low Melanin	Nitrogen	Earlier	14
12	13	Low Melanin	Unlabeled	Earlier	14
13	14	High Melanin	Carbon	Later	35
14	15	High Melanin	Unlabeled	Later	35
15	3	Low Melanin	Carbon	Later	35
16	4	Low Melanin	Nitrogen	Later	35
17	16	High Melanin	Carbon	Later	77
18	6	High Melanin	Nitrogen	Later	77
19	17	High Melanin	Unlabeled	Later	77
20	18	Low Melanin	Carbon	Later	77
21	22	Low Melanin	Nitrogen	Later	77
22	23	Low Melanin	Unlabeled	Later	77
